# The sorting receptor Rer1 controls Purkinje cell function via voltage gated sodium channels

**DOI:** 10.1038/srep41248

**Published:** 2017-01-24

**Authors:** Christina Valkova, Lutz Liebmann, Andreas Krämer, Christian A. Hübner, Christoph Kaether

**Affiliations:** 1 Leibniz Institut für Alternsforschung-Fritz Lipmann Institut, 07743 Jena, Germany; 2 Institut für Humangenetik, Universitätsklinikum Jena, Friedrich-Schiller-Universität Jena, Germany

## Abstract

Rer1 is a sorting receptor in the early secretory pathway that controls the assembly and the cell surface transport of selected multimeric membrane protein complexes. Mice with a Purkinje cell (PC) specific deletion of Rer1 showed normal polarization and differentiation of PCs and normal development of the cerebellum. However, PC-specific loss of Rer1 led to age-dependent motor deficits in beam walk, ladder climbing and gait. Analysis of brain sections revealed a specific degeneration of PCs in the anterior cerebellar lobe in old animals. Electrophysiological recordings demonstrated severe deficits in spontaneous action potential generation. Measurements of resurgent currents indicated decreased surface densities of voltage-gated sodium channels (Na_v_), but not changes in individual channels. Analysis of mice with a whole brain Rer1-deletion demonstrated a strong down-regulation of Na_v_1.6 and 1.1 in the absence of Rer1, whereas protein levels of the related Ca_v_2.1 and of K_v_3.3 and 7.2 channels were not affected. The data suggest that Rer1 controls the assembly and transport of Na_v_1.1 and 1.6, the principal sodium channels responsible for recurrent firing, in PCs.

Motor coordination in mammals is controlled by the cerebellum, a part of the hindbrain. Its cortex is composed of three layers, the molecular layer, the Purkinje cell (PC) layer and the granular layer. The PC layer consists of a single layer of PCs, the neurons with the highest synaptic input. Their axons provide the principal output of the cerebellar cortex^1^. PCs are characterized by spontaneous high-frequency firing mediated by the resurgent voltage-dependent sodium channel (Na_v_) 1.6 that localizes to the axon initial segment (AIS)^2^. Na_v_1.6, encoded by the SCN8A gene, constitutes the pore-forming large α-subunit and associates with a β2/β4 subunit and a β1/β3 subunit^3^. Another important sodium channel at the AIS of Purkinje cells is Na_v_1.1 (encoded by SCN1A)^4^, also critically involved in PC excitability^5^. Both Na_v_1.1 and 1.6 are large, glycosylated membrane proteins with four domains, each composed of 6 transmembrane domains (TM), whereas the β-subunits are single-pass TM proteins^6^. While Na_v_s are anchored to the AIS via ankyrin^7^,^8^, less is known about the mechanisms guiding proper folding, assembly and transport of Na_v_1.1 and 1.6 in the early secretory pathway. Evidence that β-subunits are involved in surface transport of Na_v_1.6 came from genetic studies^9^,^10^. In case of the closely related sodium channel in the heart, Na_v_1.5, the β1, but not the β2 subunit was shown to associate with α already in the ER^11^. How Na_v_s assemble, and which mechanisms control and guide their transport through the secretory pathway is largely unknown.

Rer1 is a sorting receptor localized to the cis-Golgi^12^,^13^. Similar to the KDEL receptor, it binds to signals in proteins destined for transient or permanent residency in the ER^14^. In contrast to the KDEL receptor, Rer1 binds to ER-retention/retrieval sequences in TM of target proteins^15^. Substrates of Rer1 in mammalian cells characterized so far are unassembled subunits of heterologous membrane protein complexes of the plasma membrane (PM)^13^,^16^,^17^ and the multi-pass membrane proteins rhodopsin^18^ and PMP22^19^. Rer1 binds to ER-retention/retrieval sequences exposed only in unassembled subunits but masked in assembled subunits^20^. Rer1 is therefore part of the quality control in the early secretory pathway that ensures that only fully assembled complexes are transported to the PM.

Using PC-specific deletion of Rer1 in mice we here show that loss of Rer1 does not affect normal polarization and differentiation of PCs, but leads to a specific defect in high-frequency firing. This defect results in a strong motor phenotype and degeneration of PCs in old mice. The underlying molecular cause is a reduction in Na_v_1.1 and 1.6 protein levels, suggesting that Rer1 is involved in the quality control of Na_v_ ion channels.

## Results

### Rer1-deficiency does not affect normal growth and differentiation of Purkinje cells

**Figure 1:**
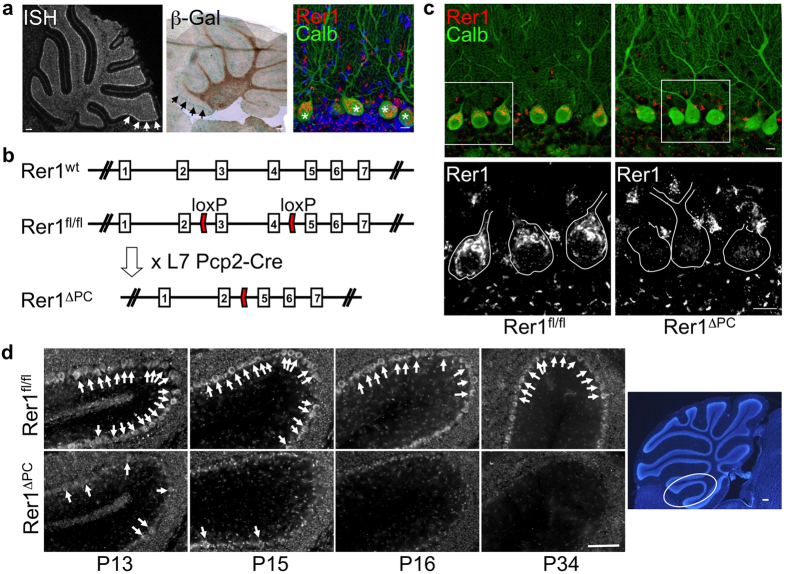
Rer1 is highly expressed in Purkinje cells (PCs) and specifically deleted in Rer1^ΔPC^ mice. (**a**) Left: radioactive *in-situ* hybridization (ISH) of a wt cerebellar section with a Rer1-specific antisense probe; middle: X-Gal staining of a cerebellar slice of a heterozygous Rer1-genetrap mouse with a β-Geo insertion in intron 3 of Rer1^13^. Arrows indicate PC layer; right: immunostaining of a cerebellar slice with Rer1 antibody (red) and calbindin (calb, green). Asterisks indicate PCs. Scalebar 150 μm (left, middle) and 10 μm (right). (**b**) Targeting strategy for conditional deletion of Rer1. Shown is the genomic structure (top), the position of the loxP sites in the Rer^fl/fl^ allele and the deleted Rer1^ΔPC^ allele after crossing with the PC-specific L7Pcp2-Cre line. (**c**) Rer1 is specifically deleted in PCs. Vibratome sections of perfused 4 months old animals with the indicated genotype were processed for immunofluorescence with antibodies against Rer1 and calbindin (Calb) and imaged by confocal microscopy. Three consecutive confocal sections were merged. In the lower panel a magnification of the Rer1 staining of the boxed area is shown. PCs are outlined. Scalebar 10 μm. (**d**) Deletion of Rer1 in PCs starts before P13 and is completed at P16. Sagittal sections from cerebella from perfused Rer1^ΔPC^ of indicated age were stained for Rer1 and images were taken from the anterior lobe at the position indicated in the overview Hoechst-stained image on the right. Scale bar 50 μm for Rer1 staining, 150 μm for Hoechst.

Rer1 is expressed throughout the rodent brain. *In-situ* hybridization and β-Gal activity from a heterozygous gene-trap allele inserted at the Rer1 locus^13^ indicated a high level of expression in cerebellar PC (Fig. 1a). Immunofluorescence staining of PC with a Rer1-specific antibody demonstrated an early Golgi-localization pattern (Fig. 1a,c, supplemental Fig. 1), as shown in other cell types^12^,^13^. Mice homozygous for the gene-trap allele inserted at the Rer1 locus are embryonic lethal at the blastocyst stage^13^. Therefore, to study the function of Rer1 *in vivo* we established a conditional knock-out mouse line by inserting loxP sites in intron 2 and 4 (Fig. 1b). Crossing these mice with L7/pcp-2:Cre, a PC specific cre-line^21^, resulted in offspring with specific Rer1 deletion in PC (Rer1^ΔPC^), but not cerebellar granule neurons or other cell types (Fig. 1c). Rer1^ΔPC^ mice are viable, fertile, have normal body weight and normal activity (data not shown). Cre-mediated deletion of Rer1 in PC was clearly observed at P13, and was completed at P16 (Fig. 1d). The cerebellum was normally developed without obvious differences in PC number or dendritic tree arborization (Fig. 1c). An indicator for proper development of PCs is the thickness of the molecular layer, because this is primarily determined by the dendritic tree of PCs^22^,^23^. No differences in mean thickness were observed in P16-25, P36 and 3 monthss old Rer1^ΔPC^ and control littermates mice (supplemental Fig. 2). Differentiation of PCs (and the cerebellum) is completed at around P21, after cre-recombination. Therefore, these data suggest that Rer1 is not involved in maturation of PCs.

### Rer1-deficiency in Purkinje cells results in severe motor deficits

**Figure 2:**
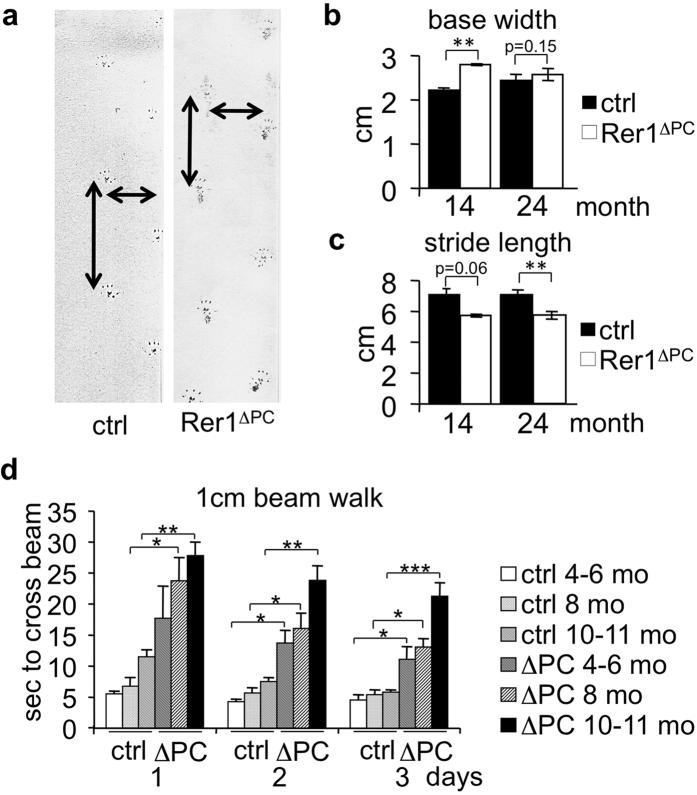
Rer1 deletion in Purkinje cells causes motor deficits. (**a–c**) Hind paws of mice of indicated age and genotype were stained with ink and the mice were allowed to walk across a paper. Base width and stride length were determined from the footprints. Displayed is the mean from 3 ctrl and 3 Rer1^ΔPC^ 14 months (mo) old mice, 5 ctrl and 5 Rer1^ΔPC^ 24 mo old mice ± SEM. (**d**) Mice of indicated age and genotype were placed on three consecutive days on a 1 cm broad, 1 m long beam and the time needed for crossing was determined. Displayed is the mean time of 4 ctrl and 4 Rer1^ΔPC^ 4-6 mo, 4 ctrl and 5 Rer1^ΔPC^ 8 mo, 6 ctrl and 6 Rer1^ΔPC^ 10–11 mo old mice ± SEM. Ctrl refers to Rer^fl/fl^/cre^−/−^ littermates.

Phenotypic analysis of young (2–3 months) mice on a Rotarod showed no difference between Rer1^ΔPC^ mice and their non-cre expressing littermates, independent of sex (supplemental Fig. 3b and not shown). However, footprint analysis of 14 and 24 months old mice revealed significant gait differences (Fig. 2a–c). When climbing a ladder, 8–12 months old Rer1^ΔPC^ mice made 2.5 times more mistakes than their littermates (2.6 ± 0.7 lapses in 12 Rer1^ΔPC^ vs. 0.04 ± 0.04 lapses in 12 control mice, t-test P = 0.006). Finally, challenging the mice on a 1 cm beam walk demonstrated that Rer1^ΔPC^ mice needed significantly more time to cross the beam, and their difficulties increased with age (Fig. 2d). Motor learning, as judged from the improvement in performing Rotarod and beam walk^24^ was not affected in Rer1^ΔPC^ mice (suppl. Fig. 3).

Taken together, the motor analysis clearly demonstrated that deletion of *Rer1* in PCs results in a severe, age-dependent gait disorder.

### Rer1-deficiency in Purkinje cells results in their age-dependent loss

**Figure 3:**
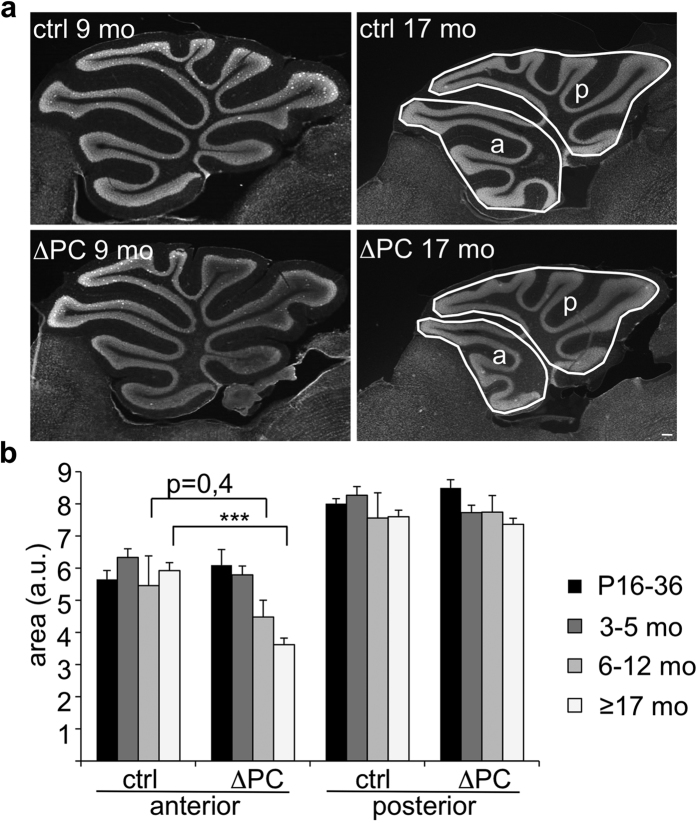
Deletion of Rer1 leads to shrinkage of the cerebellum. (**a**) Cerebella from 9 and 17 months (mo) old perfused Rer1^ΔPC^ and control littermates were sectioned and stained with Hoechst. Scalebar, 150 μm. (**b**) Anterior and posterior parts of cerebella of indicated age and genotype were marked and their area determined and displayed as mean ± SEM. n = 6 (P16-36); n = 6 (3–5 mo); n = 3 (6–12 mo) and n = 7 (≥17 mo); 3–4 sections for each mouse were analyzed. a.u., arbitrary units.

**Figure 4:**
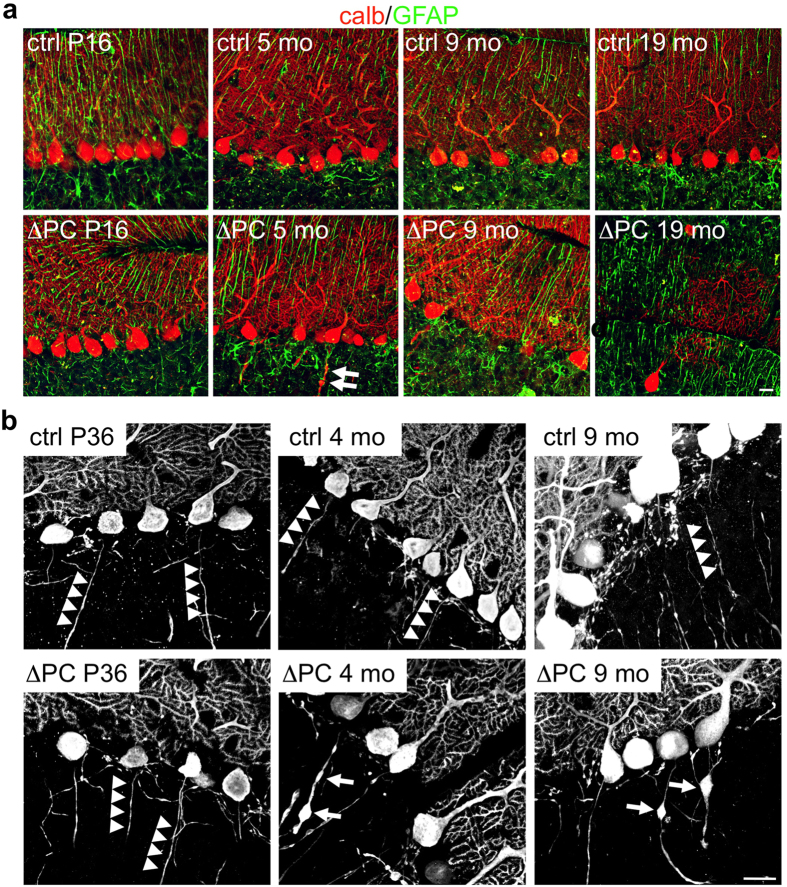
Deletion of Rer1 leads to axonal swellings and degeneration of Purkinje cells. (**a**) Cerebellar slices of mice of indicated age and phenotype were immunostained for calbindin (calb, red) and GFAP (green) and analyzed by confocal microscopy. Single confocal sections are shown. (**b**) Cerebellar slices from P36, 4 and 9 months (mo) old Rer1^ΔPC^ mice and control littermates were processed for calbindin immunofluorescence and analyzed by confocal microscopy. Shown are merges of three confocal sections. Arrowheads indicate intact axons of PCs, arrows axonal swellings. Scalebar 20 μm.

The progressive motor deficits prompted us to analyze cerebellar morphology in young and old mice of both genotypes. The overall size of the cerebellum was not different until 3–5 months of age. However, starting at the age of 6–12 months the anterior part of the cerebellum was smaller in Rer1^ΔPC^ mice. In old mice (≥17 months) the difference was statistically significant (Fig. 3a,b). Calbindin staining revealed an age-dependent, progressive loss of PCs in the anterior part starting at 5 months, where also axonal swellings were detected (Fig. 4a). Glial cells were unchanged, as indicated by GFAP co-staining (Fig. 4a). The axonal swellings, suggestive of sick or dying neurons, became visible in 4 months old Rer1^ΔPC^ mice, and were frequently detected in 9 months old Rer1^ΔPC^ mice but not in littermates (Fig. 4b). These data show that loss of Rer1 in PCs results in an age-dependent PC degeneration, which in turn leads to a selective volume reduction of the anterior cerebellum.

### Action potential generation is impaired in Rer1-deficient Purkinje cells

**Figure 5:**
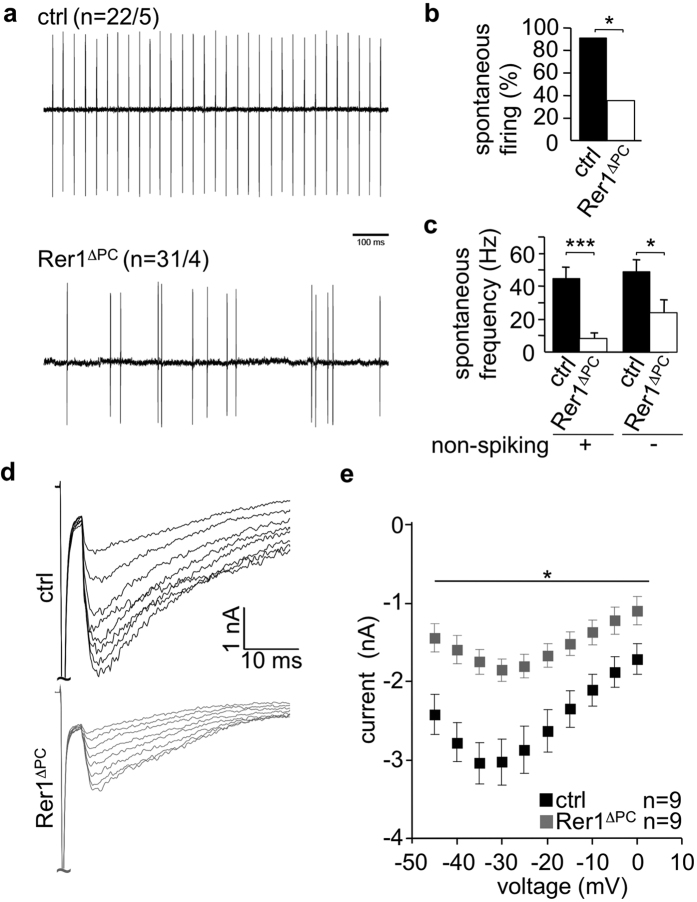
Deletion of Rer1 resulted in severe impairment of spontaneous action potential generation and decreased amplitude of resurgent currents in Purkinje cells. (**a**) PCs in cerebellar slices of 6–7 weeks old control (ctrl) and Rer1^ΔPC^ mice were patch-clamped in the loose cell-attached mode and high frequency firing was recorded. n, number of cells from 5 ctrl and 4 Rer1^ΔPC^ animals as indicated. Typical recordings for control and Rer1^ΔPC^ cells are shown, respectively. (**b**) Percentage of spontaneously firing PCs from recordings from a). (**c**) Mean firing frequency from recordings of PCs from a) including non-spiking cells (+) or excluding them (−). Non-spiking cells were defined as PCs identified by their morphology and typical low membrane resistance measured by establishment of whole cell configuration after cell attached recordings. Displayed is the mean frequency ±SD, Student’s t-test: p = 0.042; n = 20/11. Ctrl refers to Rer^fl/fl^/cre^−/−^ littermates. (**d**) Representative voltage clamp traces of resurgent sodium currents after subtraction of recordings with tetrodotoxin from recordings without tetrodotoxin recorded from PCs in cerebellar slices of 7–8 weeks old control and Rer1^ΔPC^ mice. (**e**) Current-voltage relationship of resurgent sodium currents from (**d**). n, number of cells from 5 ctrl and 5 Rer1^ΔPC^ mice; two way ANOVA, p < 0.05.

The overall morphology of PCs lacking Rer1 was not changed (Fig. 1c). To test whether their electrophysiological properties were affected, patch clamping of PCs of cerebellar slices of 6–7-weeks old Rer1^ΔPC^ and Rer1^wt^ mice was performed in the loose cell-attached mode. High-frequency firing was strongly attenuated in Rer1^ΔPC^ PCs (Fig. 5a,c; Rer1^wt^: 44.4 ± 7.4 Hz; Rer1^ΔPC^ 8.5 ± 3.1 Hz; Student’s t-test: p = 0.000013; n = 22/31). The percentage of non-spiking PCs was 7-fold increased in slices of Rer1^ΔPC^ compared to preparations of control mice (Fig. 5b; control: 9.1%; Rer1^ΔPC^ 64.5%). After exclusion of all non-spiking neurons from both groups, the analysis still revealed a significant diminished firing frequency of Rer1^ΔPC^ PCs compared with neurons from control slices (Fig. 5a,c; control: 48.8 ± 7.4 Hz; Rer1^ΔPC^ 24.1 ± 7.7 Hz; Student’s t-test: p = 0.042; n = 20/11). Next we measured resurgent currents, a characteristic feature of PCs caused by a special form of sodium channel gating^25^ in cerebellar slices of 7–8 week old Rer1^ΔPC^ mice and control littermates (Fig. 5d). The amplitude of the resurgent current was significantly reduced in Rer1^ΔPC^ PCs indicating a decrease in density of Na_v_ channels at their surface. Current-voltage relations for peak resurgent currents showed a reduced peak amplitude in Rer1^ΔPC^ mice (Fig. 5e). Interestingly, the decay constant tau at −30 mV was unchanged (11.1 ± 1.9 msec (wt) and 11.5 ± 3.03 msec (ko); Student’s t-test, p > 0.05), suggesting a decrease in density of Na_v_ channels at the cell surface, not changes in kinetic properties of individual channels. These data suggest that deficits in resurgent currents severely affect spontaneous action potential generation in Rer1^ΔPC^ PCs because of reduced amounts of Na_v_ ion channels at the AIS.

### Rer1-deficient Purkinje cells have normal axonal initial segments with reduced Na_v_1.6 levels

**Figure 6:**
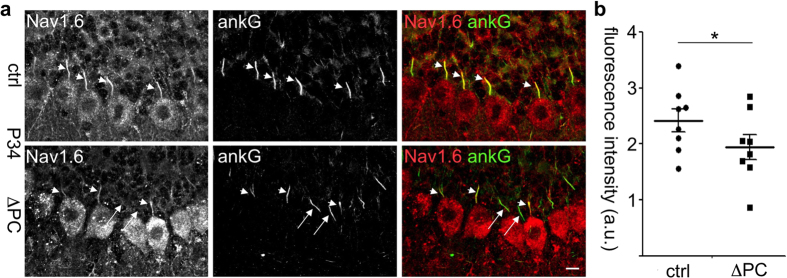
Reduced Na_v_1.6 expression at axon initial segments of Rer1-deficient Purkinje cells. (**a**) Cerebellar sections of 34 days old Rer1^ΔPC^ and control littermates mice (ctrl) were stained with antibodies against Na_v_1.6 and ankyrinG and imaged by confocal microscopy. 3 confocal sections were merged. Scalebar 10 μm. (**b**) Na_v_1.6 fluorescence intensities in the axon initial segment (AIS) were determined from sections of cerebella processed as in a). Displayed are the mean ± SEM of 243 AIS from 7 ctrl mice and 267 AIS from 8 Rer1^ΔPC^ mice. a.u., arbitrary units. Ctrl refers to Rer^fl/fl^/cre^−/−^ littermates.

**Figure 7:**
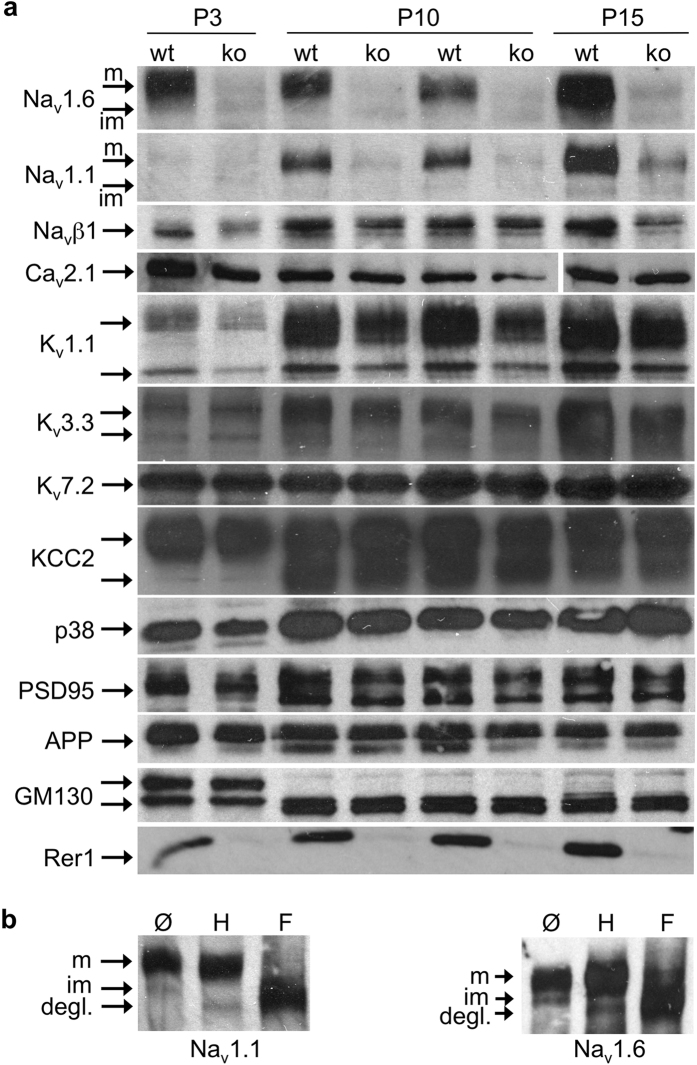
Absence of Rer1 reduces Na_v_ protein levels. (**a**) Brain lysates of Rer1^Δbrain^ (ko) and control littermates (wt) of different ages as indicated were separated by SDS-PAGE, blotted and probed with indicated antibodies. In the Ca_v_2.1 blot the irrelevant lane #7 was removed and the two last lanes flipped vertically, to match loading of the other gels. See full-length blot in the supplemental Fig. 5. P38, synaptophysin. (**b**) De-glycosylation of Na_v_1.1 and 1.6 indicates maturation status of the different bands. Brain lysates of P15 wt mice were subjected to de-glycosylation with endoglycosidase H (H) or PNGaseF (F) and processed for Western Blotting with Na_v_1.1 and 1.6 antibodies. Im, immature (endoH sensitive); m, mature (endoH resistant); degl, de-glycosylated Na_v_1.1 or 1.6 after PNGase F digest. In a, b cropped blots are shown, full-length blots are shown in supplemental Figs 5,6.

The axonal swellings, the motor phenotype as well as the high-frequency-firing deficits are similar to the phenotype of mice with a PC-specific Na_v_1.6-deletion^26^. Action potentials are generated at the AIS, where Na_v_1.6 is enriched^2^. We therefore tested whether the structure of the AIS or its molecular composition is dependent on Rer1 and performed co-immunostainings with ankyrin G and Na_v_1.6 (Fig. 6a, for specificity of Na_v_1.6 antibody see supplemental Fig. 4). No gross difference was observed in ankyrin G and Na_v_1.6 distribution between Rer1^ΔPC^ mice and control littermates and the average AIS-length didn’t differ between the two genotypes (14.2 ± 0.3 μm in control, 14.1 ± 0.3 μm in Rer1^ΔPC^ mice; n = 243 AIS from 7 ctrl mice and n = 267 AIS from 8 Rer1^ΔPC^ mice). However, the average intensity of Na_v_1.6 at the AIS was significantly diminished in PCs of Rer1^ΔPC^ mice, sometimes below the detection limit (Fig. 6a, long arrows). From 152 AIS from control littermates (n = 6) 9,95 ± 1,6% had very low or undetectable Nav1.6 immunostaining. In contrast, from 169 AIS from Rer1^ΔPC^ mice (n = 6) almost three times more AIS (26,3 ± 6%) had very low or undetectable Nav1.6 immunostaining. Consistently, cerebellar lysates of Rer1^ΔPC^ mice had slightly reduced levels of Na_v_1.6 at P23, before overt loss of PCs (not shown). Since PC only constitute a minor fraction of the cerebellum, and because of the specificity of the deletion, large differences in total cerebellar lysates are unlikely. We therefore made use of another mouse line in which Rer1 is deleted in the whole brain using nestin-cre (Rer^Δbrain^). These mice die early postnatally. Brain lysates from P3, P10 and P15 Rer^Δbrain^ mice showed almost complete absence of Rer1, demonstrating successful deletion (Fig. 7a). Rer1 deficiency does not change general brain architecture, indicated by the unchanged protein levels of synaptophysin (p38), PSD95, APP and the early Golgi marker GM130 in brain lysates (Fig. 7a) and by histological analysis (not shown). However, Rer^Δbrain^ mice displayed strong reductions in Na_v_1.6 expression levels (Fig. 7a). Also the protein levels of the closely related Na_v_1.1 are strongly reduced. In contrast, protein levels of an abundant calcium channel from the same ion channel superfamily, Ca_v_2.1, and of three tested potassium channels K_v_1.1, 3.3 and 7.2, as well as the levels of a K-Cl Cotransporter KCC2, are not changed in Rer^Δbrain^ mice. Protein levels of the Na_v_β1 subunit were only slightly reduced in Rer^Δbrain^ mice (Fig. 7a). Na_v_1.1 and 1.6 are complex glycosylated. De-glycosylation using endoglycosidase H (endoH, cleaves only immature sugars on ER/early Golgi-resident proteins) and PNGase F (F, removes all N-linked sugars) showed that the higher, prominent band in Na_v_1.1 and Na_v_1.6 is endoH resistant (Fig. 7b). This fraction corresponds to the mature channel at the PM, while the weaker, faster migrating band is endoH sensitive, corresponding to the immature fraction of the channel in the ER/early Golgi that is not yet fully folded and/or assembled (Fig. 7b). Only the mature Na_v_1.1 and 1.6 levels are strongly reduced in the absence of Rer1 and no accumulation of immature channels is observed (Fig. 7a). This suggests that in the absence of Rer1 misfolded and/or unassembled Na_v_1.1 and 1.6 escape the ER quality control and are degraded in later compartments, similar to the acetylcholine receptor^13^. Rer1 is not involved in controlling Ca_v_ or K_v_ channels and not or only to a minor degree in β1.

Taken together, we show that loss of Rer1 in PC elicits a very specific phenotype with a loss of functionality of PCs causing severe motor deficits and age-dependent degeneration. Rer1 is essential for the key property of PCs, their ability to generate high-frequency action potentials, by specifically controlling protein levels of mature Na_v_ ion channels.

## Discussion

The PC, and a neuron in general, is an incredibly complex information processing entity. It only functions properly if a plethora of proteins and mechanisms interact like cogs mesh in a gearbox. For example, for generation of the resurgent Na^+^ current that is essential for action potentials in PCs, a specific voltage-dependent Na-channel, the Na_v_1.6, with its multiple TM domains needs to be co-translationally inserted into the ER, properly folded, assembled with the correct β-subunit(s), transported through the Golgi, sorted into vesicles destined for the AIS, inserted there, and fulfill its function as an ion channel. We here show that a disturbance at one or a few of these steps has dramatic consequences. Loss of Rer1, and thus disturbance of the quality control system that ensures proper folding and assembly of multimeric complexes, leads to lower amounts of mature Na_v_1.1 and 1.6 in PCs. This results in almost complete failure of the constant firing of action potentials, which in turn results in the severe motor phenotypes we observed in older mice. PCs fail to generate action potentials at a very young age, and maybe were never able to do so. We could not determine the earliest time point where motor problems can be detected. Yet, the development of motor phenotypes is clearly age-dependent, similar to other cerebellar mouse mutants^27^, probably because of initial compensation for loss of function. In contrast, the early complete loss of PCs at P15-30 in the *Pcd* mice results in early ataxia^28^,^29^ and problems on the Rotarod already at P20^30^. In mice with a PC-specific deletion of Na_v_1.6 early ataxia and impaired Rotarod performance was observed at 6–8 wks^26^. Why our Rer1^ΔPC^ do not show motor coordination problems on the Rotarod is unclear, but could be due to the rather insensitive Rotarod^29^ or because of residual Na_v_1.1 and 1.6 that correctly assemble and make it to the AIS in the absence of Rer1. Interestingly, motor learning as assessed from the learning curves on Rotarod and beam walk was not affected by the deletion of Rer1, similar to the *tambaleante* mice, where PCs are lost after 4–6 months^24^. However, for a detailed analysis of motor learning eyeblink classical conditioning needs to be performed, as for example described in ref. 31.

PCs without Rer1 are normally developed and maintained to young adulthood. However, they start to degenerate in adult mice, maybe as a consequence of the loss of functionality. This degeneration leads to reduction of cerebellar size in old mice and probably contributes to the severe motor phenotypes. Interestingly, the degeneration affects mainly the anterior lobe, although Rer1 is deleted in the whole cerebellum. The underlying cause is not known, but patterned or area-selective cell death of PC has been frequently observed (reviewed in ref. 32). The most prominent pattern in PCs is the pattern of zebrin-positive and zebrin-negative zones, with the PCs in the respective zone having distinct electrophysiological properties^33^. Expression of Rer1 is similar in PCs from all areas of the cerebellum and is not patterned. Unlike for example in model mouse lines of Niemann-Pick Type C disease^34^,^35^, in Rer1^ΔPC^ mice PC degeneration seems uniform and not patterned. However, since in the anterior lobe the zebrin-negative areas dominate (where we see the prominent PC loss), a potential correlation has to be analyzed in more detail in future studies.

The evidence that disturbances in voltage-gated sodium channels Na_v_1.1 and 1.6 homeostasis are the main cause for the observed phenotype is several fold. PC-specific deletion of Na_v_1.6^26^, loss-of-function-mutation in Na_v_1.1^5^ and deletion of Rer1 (this study) in mice caused very similar phenotypes. All models displayed severe motor deficits caused by a strongly reduced firing rate of PCs. Both Levin *et al*.^26^ and we observed swellings in axons of PCs and we additionally observed PC degeneration and cerebellar shrinkage in older mice, which was not analyzed by Levin *et al*.^26^. Expression levels of Na_v_1.1 and 1.6 are strongly reduced in the absence of Rer1. The protein levels of β1 are only slightly reduced, suggesting it is not a direct substrate of Rer1. In addition, it was shown that loss of *scn1b* (coding for the β1 subunit) leads to a reduction in Na_v_1.6 with a concomitant up-regulation of Na_v_1.1^36^, which we did not observe in our mice. This argues against Rer1 acting via β1 on Na_v_1.1 and 1.6. The β2-subunit does not seem to be involved in assembly of Na_v_. Deletion of its gene, *scn2b*, causes only mild phenotypes unrelated to the motor deficits observed here^37^. Rer1 seems to act specifically on Na_v_ channels, not on closely related Ca_v_ and on K_v_ channels.

A number of substrates for Rer1 have been identified^13^,^16^,^17^,^18^,^19^, but *in vivo* evidence for Rer1 function in mammals was scarce. We here provide *in vivo* evidence of the role of Rer1 in a specific cell type, the murine PC. Importantly, the deletion of Rer1 in our model occurs at a stage where the PCs (and the cerebellum) are not yet fully developed. Yet, loss of Rer1 had no observable effect on the thickness of the molecular layer, indicative of a normally developed dendritic tree^22^,^23^. Altogether, this strongly suggests that major developmental pathways are not affected and that Rer1 is not a general sorting receptor. Instead, Rer1 probably has only a very limited number of substrates. Specifically in PC the most prominent substrate seems to be Na_v_s, but in the current study we cannot rule out that there are additional substrates of Rer1 in PC. Using morpholinos and siRNA against Rer1 in zebrafish and mammalian cells, respectively, resulted in shorter primary cilia and developmental defects^38^. We find no evidence for a developmental role of Rer1 in PC, but cannot rule out that a deletion at an earlier time point would have an effect. Primary cilia in PCs were difficult to detect in our hands, but primary cilia length was not changed in the hippocampus of brains with forebrain-specific Rer1 deletion (by CamKII-cre; mean ciliary length in dendrate gyrus was 5.5 ± 0.2 pixels in wt, 6.0 ± 0.3 pixels in KO mice, in CA1 region 10.4 ± 0.5 pixels in wt, 11.4 ± 0,4 pixels in KO mice). This suggested Rer1 is not involved in cilia function in mammalian postnatal brain, but might be important for cilia formation in different organs or organisms.

Further work is needed to characterize the exact mechanism of Rer1 involvement in Na_v_ biogenesis. In analogy to the role of Rer1 in acetylcholine receptor assembly^13^, we hypothesize that the assembly rates of Na_v_ are reduced. Free subunits, normally returned by Rer1, escape the ER/cis-Golgi and get degraded, probably in lysosomes, and this results in the reduced total levels of mature Na_v_.

Mutations in *SCN1A* (encoding Na_v_1.1) are among the most frequent mutations in epilepsy^39^,^40^ and mutations in *SCNA8A*, the gene encoding Na_v_1.6, were found in patients with epileptic encephalopathy and mental disabilities^41^. Mutations leading to increased channel activity might lead to seizures, whereas reduced activity might lead to intellectual disabilities^41^. Not surprisingly, Na_v_s are major drug targets^42^. Future work has to elucidate to what extent Rer1 controls Na_v_ channels also in other brain areas besides the cerebellum and whether it might be a novel therapeutic target. Increasing Rer1 levels or stabilizing its interaction with Na_v_ might overcome deficits caused by loss-of-function or hypomorphic mutations, similar to other rescue approaches^43^,^44^. A partial rescue might be sufficient, as has been shown in the case of mutations in Na_v_1.1 where a partial rescue by lowering temperature or co-expressing binding proteins resulted in a constitutive gain-of-function^43^. In case of over-active mutations reducing Rer1 levels or Na_v_1.6 interactions might be beneficial.

In conclusion, we here present the first *in vivo* data how a member of the ER/Golgi quality control machinery, the sorting receptor Rer1, controls a specific class of voltage-gated sodium ion channels that are essential for the electrical properties of cerebellar Purkinje cells.

## Methods

### Antibodies

The following antibodies were used. Anti-Rer1, rabbit polyclonal SA5457 and SPY008^13^; anti-calbindin-D-28K, mouse monoclonal (C-9848), anti-MAP2 mouse monoclonal M4403 and anti-GFAP, rabbit polyclonal (G-9269), all from Sigma; anti-Nav1.6 (ASC-009), anti-Nav1.1 (ASC-001), anti-Cav2.1 (ACC-001), anti-Kv1.1 (APC-009), anti-Kv3.3 (APC-102) and anti-Kv7.2 (APC-050), all rabbit polyclonal from Alomone Labs; anti-ankyrin G mouse monoclonal (sc-12719), Santa Cruz Biotechnology; anti-Scn1b, rabbit polyclonal (AP53815 PU-N), Acris; anti-APP, mouse monoclonal (MAB348), Millipore; anti-PSD-95, mouse monoclonal (6G6-1C9, VAM-PS002), Assay Designs; anti-KCC2, rabbit polyclonal (07–432) and anti-synaptophysin mouse monoclonal (MAB368) both from Millipore, anti-GM130, mouse monoclonal (610822) BD Transduction Laboratories; anti-Sstr3, rabbit polyclonal antibody kindly obtained from Stefan Schulz, Jena^45^; Alexa Fluor-labeled secondary antibodies, Molecular Probes, HRP-conjugated secondary antibodies, Promega.

### Targeted inactivation of murine *Rer1*

The Rer1 conditional KO mice were generated by TaconicArtemis (now Taconic Biosciences GmbH), Cologne, Germany. Exons 3–4 were flanked by LoxP sites. Positive selection markers were flanked by FRT (NeoR) and F3 (PuroR) sites and inserted into intron 2 and intron 4, respectively. A targeting vector was generated using BAC clones from a C57BL/6J RPCI-23 BAC library and transfected into TaconicArtemis C57BL/6N Tac ES cell line. After isolation of homologous recombinant clones using positive (Neomycin and Puromycin resistances) and negative (Thymidine kinase - Tk) selections, the conditional KO allele was obtained after Flp-mediated removal of selection markers. Chimeric mice were generated by blastocyst injection, germline transmitters selected and kept in C57Bl/6 background. For conditional inactivation of Rer1, Rer1^fl/fl^ mice were crossed with L7/pcp-2:Cre mice (for specific Purkinje cell deletion^21^) or with nestin-cre mice (for CNS-specific deletion^46^) or with CamKII-cre (for forebrain-specific deletion^47^).

### Mice

All animal experiments were conducted according to the German animal welfare legislation and approved by the Thüringer Landesverwaltungsamt (reg. nr 03-008/12). Mice were fed *ad libitum* with standard laboratory chow and water in ventilated cages under a 12 h light/dark cycle.

### Motor coordination tests

#### Gait analysis

Female mouse hind paws were painted with non-toxic, water-soluble ink and footprint patterns were analyzed using a runway (80 cm ×10.5 cm wide) with white paper on the bottom. Five consecutive strides were measured from three runs of each animal.

#### Ladder-climbing

Female mice were habituated to climb to their home cage on a 1,1 m long ladder inclined to 22,5° angle with steps 2 cm apart and the average number of lapses was counted from video-recordings from 3 runs per mice. Male mice behaved identically (data not shown).

#### Beam walking

Female mice were trained to run along a 1 m long beam (3 cm thick) to their home cage. The test was performed on three consecutive days on a 2 cm thick beam followed by three consecutive days on a 1 cm thick beam with two runs each day. The mice were video-taped and the time to cross the beam and the number of foot slips were measured. Male mice behaved identically (data not shown).

### Electrophysiology

#### Slice preparation for electrophysiological recordings

350-μm-thick brain slices were prepared from 6–7 weeks old mice of either sex and equilibrated in aCSF (in mM): 120 NaCl, 3 KCl, 1.3 MgSO_4_, 1.25 NaH_2_PO_4_, 2.5 CaCl_2_, 10 D-glucose, 25.0 NaHCO_3_, gassed with 95% O_2_/5% CO_2_, pH 7.3 at room temperature for at least 1 h as described previously^48^.

#### Patch clamp recordings

Coronal cerebellar slices were placed in a submerged recording chamber mounted on an upright microscope (BX51WI, Olympus). Slices were continuously superfused with gassed aCSF (2–3 ml/min, 32 °C, pH 7.3). Loose-patch cell attached recordings (seal resistance < 1 GΩ) were performed under visual control with a 40x water-immersion DIC objective. Purkinje-neurons with typical dendritic trees and large cell bodies were selected for recordings using a patch-clamp amplifier (Multiclamp 700B, Molecular Devices). Responses were low-pass filtered at 4 kHz and digitized at 20 kHz (Digidata 1440, Molecular Devices). All data were acquired, stored, and analyzed using pClamp 10.2 and Clampfit 10.2 (Molecular Devices). Patch pipettes with an impedance of 3–4 MΩ were pulled from borosilicate glass (outer diameter 1.5 mm, Science Products GmbH) on a Sutter Instrument micropipette puller (P-97) and filled with aCSF. Spike frequencies were calculated for each cell from recordings with a minimal duration of 1 min. For voltage clamp recordings of resurgent sodium currents from Purkinje neurons of acute cerebellar slices a slightly modified protocol was used as described previously^49^. As extracellular solution served a modified aCSF (in mM): 110 NaCl, 3 KCl, 1.3 MgSO_4_, 1.25 NaH_2_PO_4_, 2.5 CaCl_2_, 10 TEA-chloride (tetraethylammonium chloride), 0.04 CdCl_2_, 10 D-glucose and 25 NaHCO_3_, gassed with 95% O_2_/5%CO_2_, pH7.3. Patch pipettes were filled with (in mM): 135 CSF, 1 NaCl, 3 KCl, 2 Mg-ATP, 0.5 Na-GTP, 10 HEPES, 5 EGTA adjusted to pH 7.3 with CsOH. After whole-cell configuration was established, cells were recorded. From a holding potential of −90 mV cells were depolarized to + 30 mV for 3 ms followed by steps from −50 mV to 0 mV with an increment of 5 mV. For determination of the resurgent current amplitudes 1 μM tetrodotoxin was applied and the protocol was repeated and subtracted from the initial recording. The time constant (τ) was determined by monoexponential fitting.

### Immunohistochemistry

Mice of either sex were anesthetized with Isoflurane and perfused transcardially with 4% paraformaldehyde (PFA) in phosphate-buffered saline (PBS). The brain tissue was post-fixed in 4% PFA/PBS overnight at 4° C, washed in PBS and embedded in 4% agarose. 50 μm thick sagittal brain sections were obtained using a vibratom and were stored in PBS with 0.05% NaN_3_ at 4° C.

For immunostainings vibratom sections were mounted on Superfrost^®^ Plus microscope slides (ThermoScientific) and air dried. For antigen retrieval the sections were placed in pre-warmed 10 mM sodium citrate buffer pH 6.0, briefly boiled and incubated for 10 min at sub-boiling temperature (10%) in a microwave followed by 30 min at room temperature. After washing with PBS the sections were incubated for 1 h in blocking solution (5% normal goat serum, 1% BSA and 0,4% Triton X-100 in PBS) and then overnight at 4° C with primary antibodies in blocking solution, washed with PBS, then incubated for 1 hour at RT with Alexa Fluor-conjugated secondary antibodies (1:1000) and Hoechst and after wash with PBS were mounted in Fluoromount (F4680, Sigma).

### *In-situ* hybridization

Radioactive *in-situ* hybridization using a full-length mouse Rer1 cDNA in pBluescript was performed as described^50^.

### Immunoblotting

Brain tissues of mice of either sex were lysed in STEN-lysis buffer (50 mM Tris pH 7.6, 150 mM NaCl, 2 mM EDTA, 1% NP40 and protease inhibitor mix) or in Complexolyte 48 buffer (Logopharm) with protease inhibitor mix using Precellys beads (1,4 mm) and the Precellys 24 lysis and homogenization device for 2 × 15 sec at 5000 rpm (PeqLab). Proteins were separated on 6% SDS-PAGE gels (for detection of Na_v_, Ca_v_), 10% for detection of Kv, KCC2 or 12% gels (for detection of Rer1, Na_v_β1, PSD-95, APP, p38 and GM130) and transferred to PVDF membranes. Membranes were cut at appropriate positions and blotted with antibodies as indicated.

### Microscopy and image quantifications

For higher resolution immunhistochemistry a Zeiss 510 Meta LSM equipped with 25x/0.8 NA and 40x/1.3NA objectives was used. For overviews of cerebellar morphology an Olympus AX70 with 4x/0.10 NA objective was used. For the primary cilia length and molecular layer thickness determination a Zeiss Observer Z1 equipped with 10x/0.3NA and 20x/0.8NA objective and an Apotome slider was used. The thickness of the molecular layer was measured on 2D images of calbindin-immunostainings of the cerebellum along the III-IV and VIII-IX lobes at six positions at each lobe in 2–3 midsagittal sections per mouse (3 wt and 3 Rer1^ΔPC^ mice). For measurement of cilia length 3 sagittal sections per mouse (3 wt and 3 Rer1^ΔFN^ mice) were stained with anti-sstr3 antibody^45^. ApoTome sections from CA1 and DG regions of the hippocampus were taken and 3D reconstructions of were made using ZEN software. The length of cilia (in pixels) was measured using the polygon curve tool. At least 200 cilia were measured per region of the hippocampus. The anterior and posterior cerebellar areas were measured on 2D images of Hoechst-stainings of three mid-sagittal sections per mouse (3 ctrl and 3 Rer1^ΔPC^ mice). Using NIH ImageJ software the anterior area was measured within a line drawn around the lobes I-V^51^ that are anterior from the primary fissure and respectively the posterior area was measured within a line drown around the lobes VI-IX that are posterior from the primary fissure. For measurement of the axon initial segment confocal images from midsagittal sections stained with anti-ankyrin G and anti-Nav1.6 antibodies were obtained using identical settings. Image stacks were converted into a maximal intensity Z-axis projection and measurements were performed. The fluorescence intensity of Na_v_1.6 was measured using NIH ImageJ software by drawing a segmented line along the length of the Ankyrin G signal at the AIS. The background fluorescence was subtracted based on a 1 μm segment distal to the AIS. The AIS fluorescence density was calculated as integrated density divided by the AIS length.

### Statistical analysis

Data are always presented as mean + /− SEM and the numbers of experiments or mice (n) are indicated. The unpaired Student’s t-test was applied to evaluate differences between experimental groups (GraphPad Prism software). P-values ≤ 0.05 were considered statistically significant. One asterisk P ≤ 0.05; two asterisks P ≤ 0.01; three asterisks P ≤ 0.001.

## Additional Information

**How to cite this article**: Valkova, C. *et al*. The sorting receptor Rer1 controls Purkinje cell function via voltage gated sodium channels. *Sci. Rep.* **7**, 41248; doi: 10.1038/srep41248 (2017).

**Publisher's note:** Springer Nature remains neutral with regard to jurisdictional claims in published maps and institutional affiliations.

## Supplementary Material

Supplementary Figures
