# Supplementary material for: The sorting receptor Rer1 controls Purkinje cell function via voltage gated sodium channels
Source: Sci Rep. 2017 Jan 24;7:41248. doi: 10.1038/srep41248 (PMC5259745; doi:10.1038/srep41248)
Supplement: Supplementary Figures [file srep41248-s1.pdf]

The sorting receptor Rer1 controls Purkinje cell function via voltage gated sodium channels

Christina Valkova[1], Lutz Liebmann[2], Andreas Krämer[1], Christian Hübner[2] and Christoph Kaether[1#]

[1]Leibniz Institut für Alternsforschung-Fritz Lipmann Institut, 07743 Jena, Germany, [2]Institut für Humangenetik, Universitätsklinikum Jena, Friedrich-Schiller-Universität Jena, Germany

Supplemental information

Supplemental figures 1-6

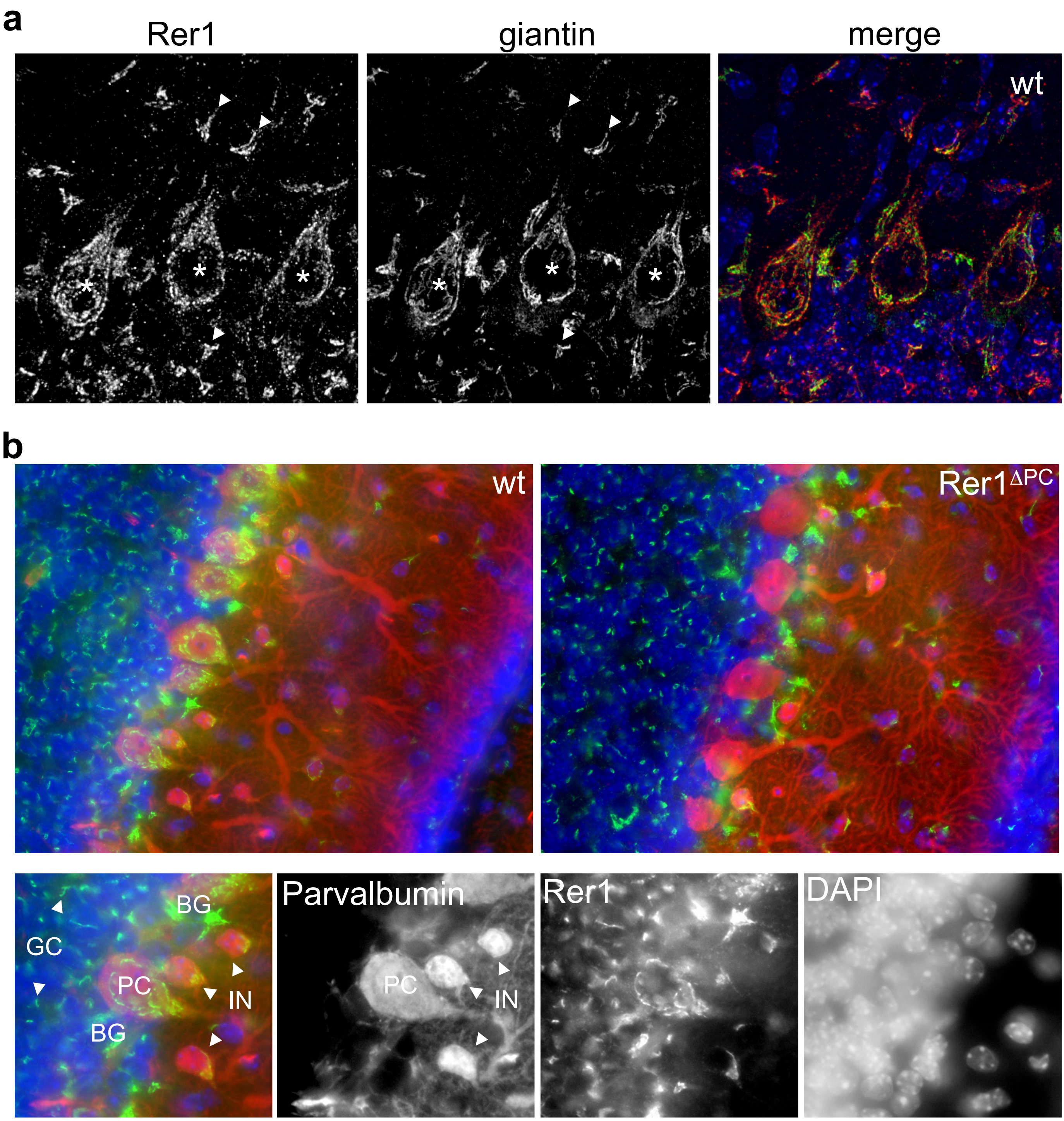

Supplemental Figure 1: Rer1 localizes to the Golgi of several cell types in the cerebellum. Cerebellar vibratome sections of mice with indicated genotype were immunostained with antibodies against Rer1 and giantin, a Golgi marker (a) or Parvalbumin, a marker of GABA-ergic neurons (b), and imaged by confocal microscopy. a) Purkinje cells (asterisks) as well as granule cells and cells in the molecular layer (arrows) show co-localization of Rer1 and giantin. b) Rer1 is expressed in granule cells (GC), Purkinje cells (PC), interneurons (IN) and Parvalbumin negative (and Calbindin negative, Fig. 1) cells, probably Bergman Glial cells (BG). Scalebar 10 μm.

a

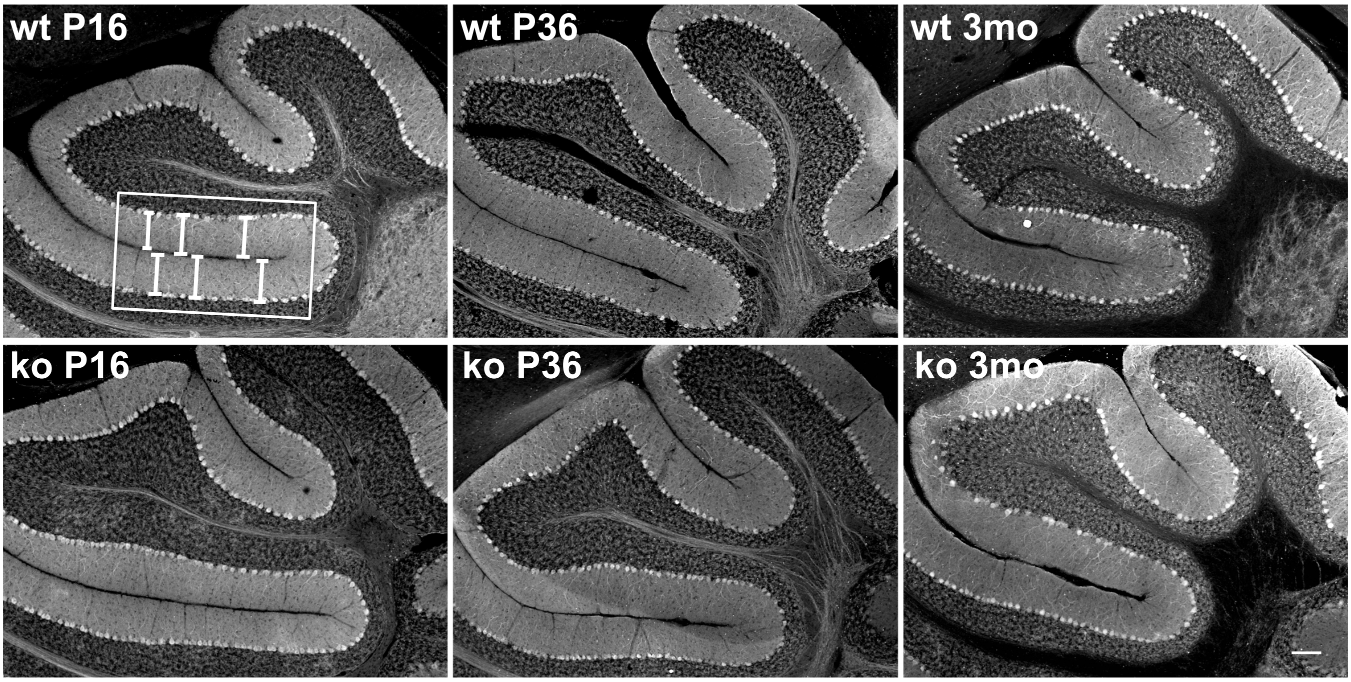

b

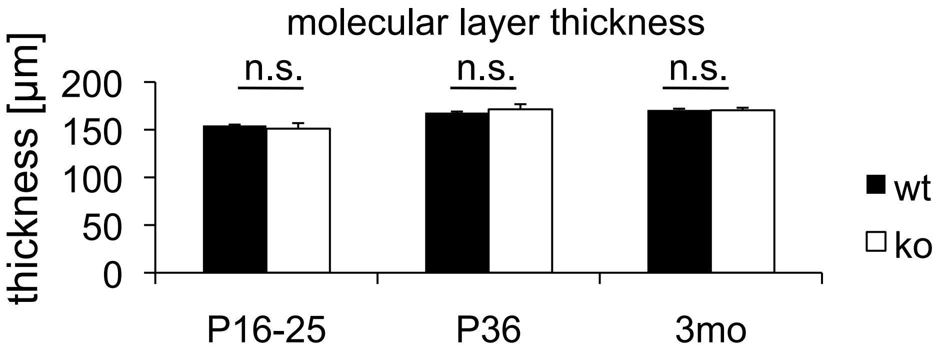

Supplemental Figure 2: Thickness of molecular layer as indication for PC development is not changed in young Rer1$^{\Delta PC}$ mice. a) Midsagittal sections of cerebelli of indicated age and genotype. Shown are examples of anterior parts of the cerebellum. Measurements were made in lobe III-IV (boxed in top left image). b) Quantification of molecular layer thickness. Displayed are the mean values of 3 wt and 3 ko animals (2-3 sections/animal. 12 measurements per section: 6 anterior (lobe III-IV), 6 posterior (lobe VIII-IX). Error bar SEM. No differences were observed between genotypes. Scale bar 100 µm.

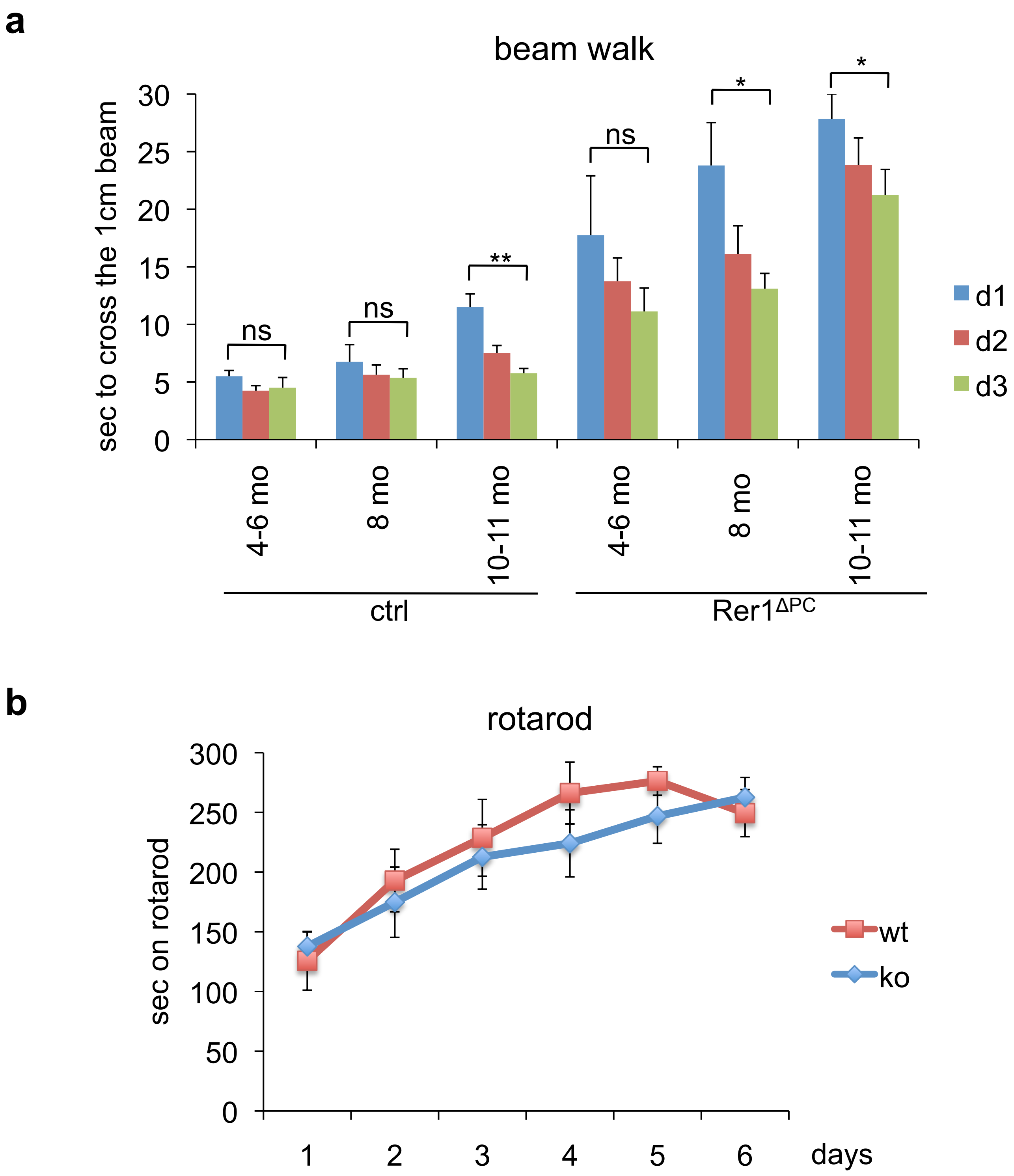

Supplemental Figure 3: Loss of Rer1 in PC does not cause motor learning problems. a) Data from the 1cm beam walk experiment in Fig. 2d were rearranged to show the improvement in beam walk crossing in Rer1$^{\Delta PC}$ mice at all ages tested. d, day; mo, month. The stronger learning effect of the Rer1$^{\Delta PC}$ mice can be explained by the already very good starting performance of the control mice at 4-6 and 8 month. b) shows the learning curves of 2-3 months old wt and Rer1$^{\Delta PC}$ (ko) mice on a rotarod on 6 consecutive days. No differences between wt and ko were observed.

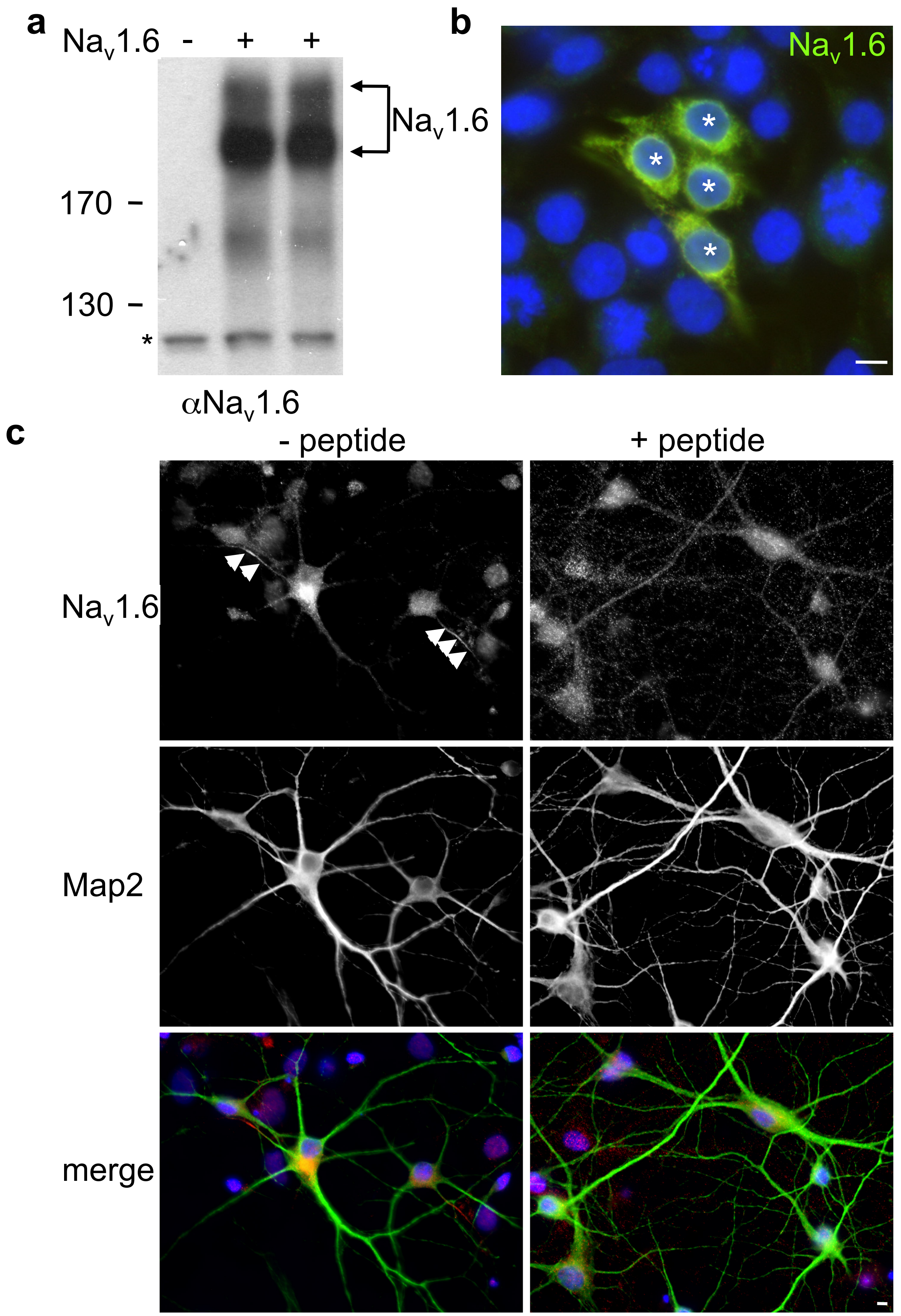

Supplemental Figure 4: The Nav1.6 antibody specifically detects Nav1.6. a, b) HEK293 cells transfected with plasmid pRc/CMV (A)-Scn8a (kindly provided by Alan Goldin) were lysed, separated by SDS-PAGE and blotted with anti-Nav1.6 antibody (a) or fixed and processed for immunofluorescence with anti-Nav1.6 antibody (b). Asterisk in (a) indicates an unspecific band, in (b) transfected cells. No band at the expected molecular weight and no staining is detected in untransfected cells. c) Primary rat hippocampal cultures at DIV 14 were fixed and processed for immunofluorescence with anti-Nav1.6 antibody in the presence and absence of the peptide used for immunization and with Map2 as dendritic marker. Arrowheads depict the AIS with specific Nav1.6 staining (left panel) that is competed by adding the peptide (right panel). Scalebar 10 μm.

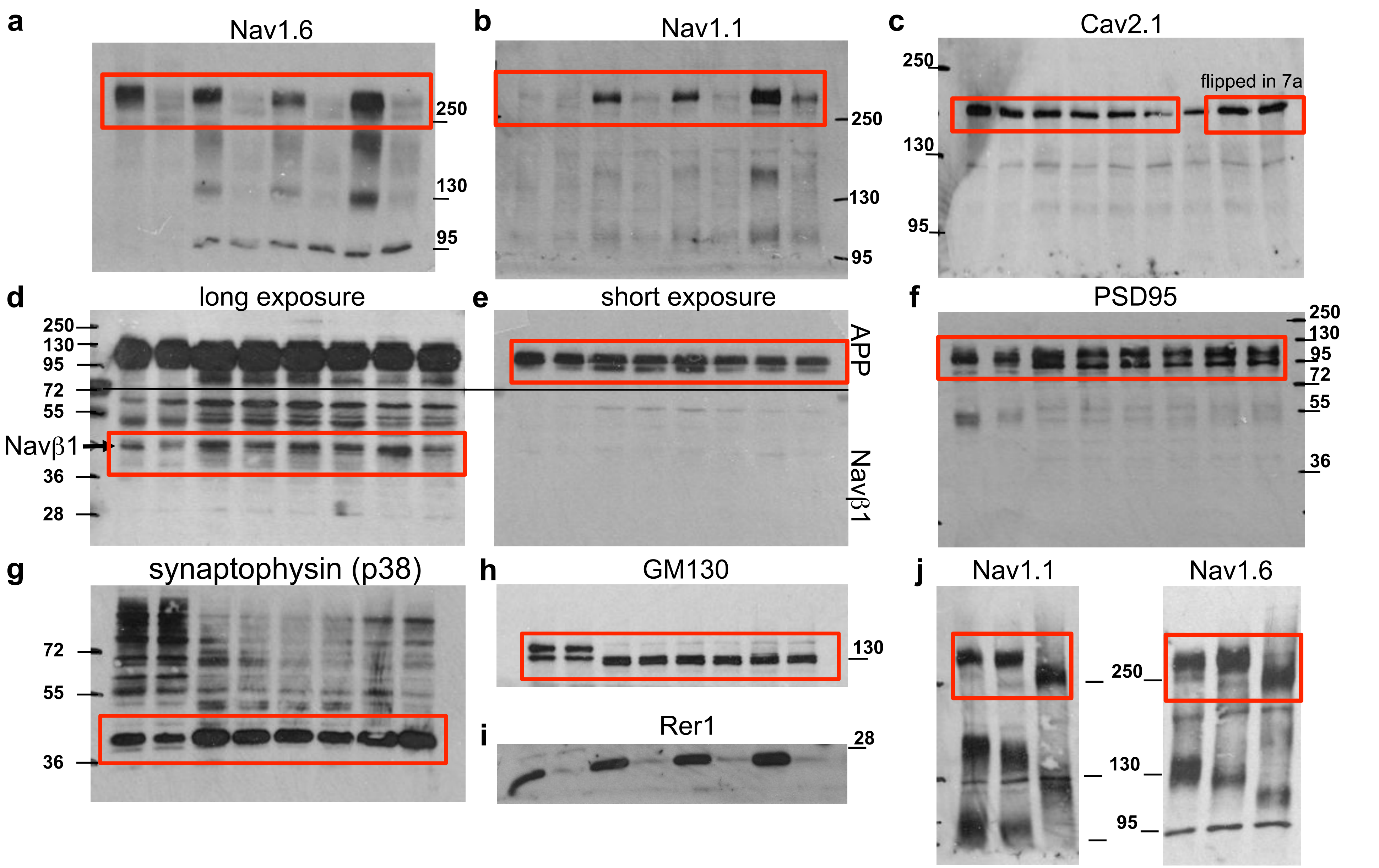

Supplemental Figure 5: Full-length blots corresponding to Fig. 7. Indicated parts (red box) of a-i) are shown in Fig. 7a. From blot c) line 7 was removed and the last two lanes flipped, to match loading of the other blot. Indicated parts in j) are shown in Fig. 7b. In d,e) the upper half, indicated by black line, was probed for APP, the lower for Navβ1. Different exposure times are shown. In h,i) lower and upper parts, respectively, were cut and probed with different antibodies, not used here. MW indicated in kDa.

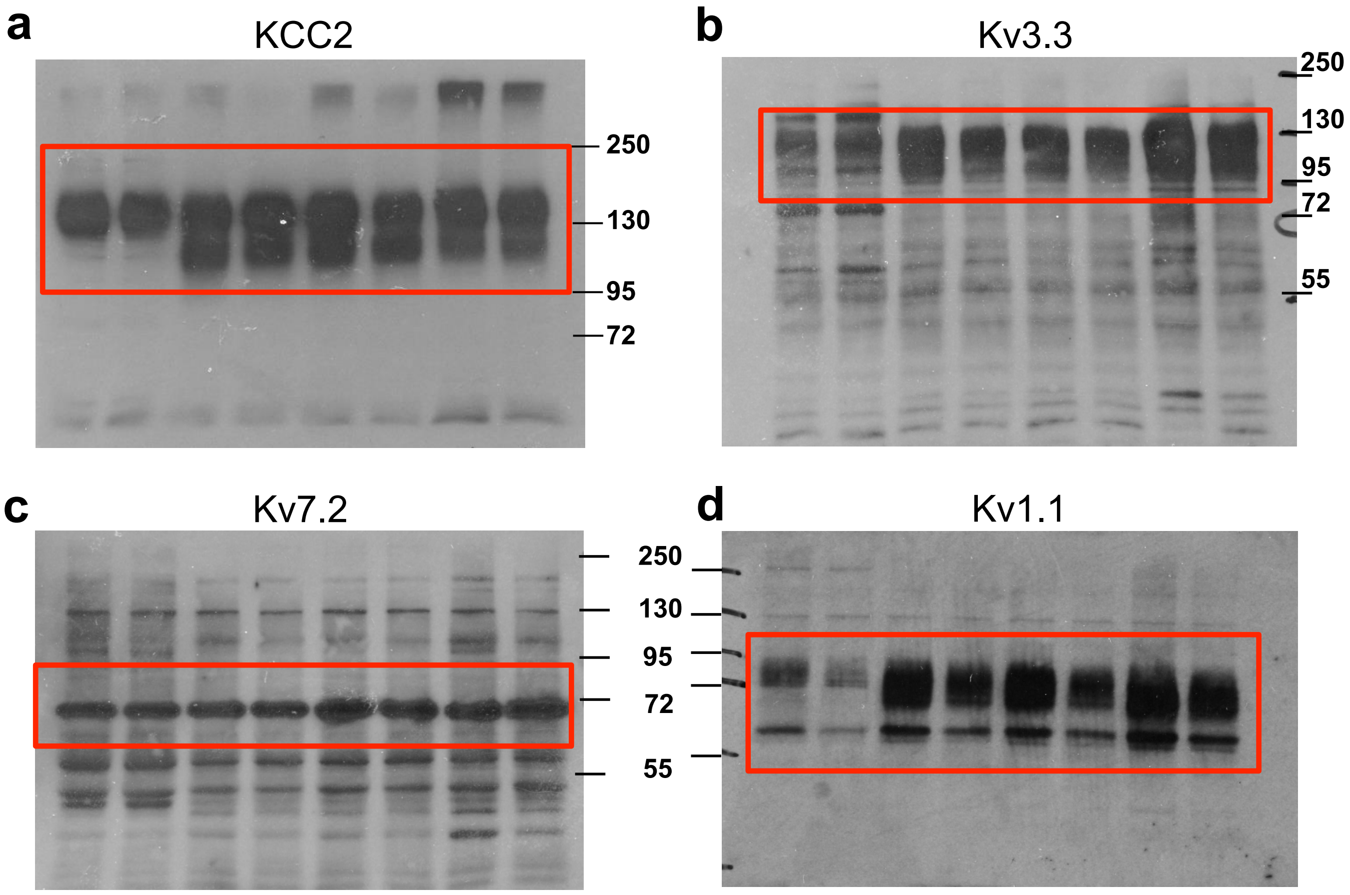

Supplemental Figure 6: Full-length blots corresponding to Fig. 7a. Indicated parts (red box) of a-d) are shown in Fig. 7a. MW indicated in kDa.
